# Assessment on the oil accumulation by knockdown of triacylglycerol lipase in the oleaginous diatom *Fistulifera solaris*

**DOI:** 10.1038/s41598-021-00453-w

**Published:** 2021-10-22

**Authors:** Yoshiaki Maeda, Kahori Watanabe, Marshila Kaha, Yusuke Yabu, Tomoko Yoshino, Mitsufumi Matsumoto, Tsuyoshi Tanaka

**Affiliations:** 1 Division of Biotechnology and Life Science, Institute of Engineering, Tokyo University of Agriculture and Technology, 2-24-16 Naka-cho, Koganei, Tokyo 184-8588 Japan; 2 Biotechnology Laboratory, Electric Power Development CO., Ltd., 1, Yanagisaki-machi, Wakamatsu-ku, Kitakyushu, Fukuoka, 808-0111 Japan

**Keywords:** Biotechnology, Molecular biology

## Abstract

Microalgae are promising producers of biofuel due to higher accumulation of triacylglycerol (TAG). However, further improvement of the lipid metabolism is critical for feasible application of microalgae in industrial production of biofuel. Suppression of lipid degradation pathways is a promising way to remarkably increase the lipid production in model diatoms. In this study, we established an antisense-based knockdown (KD) technique in the marine oleaginous diatom, *Fistulifera solaris*. This species has a capability to accumulate high content of lipids. *Tgl1* KD showed positive impact on cell growth and lipid accumulation in conventional culture in f/2 medium, resulting in higher oil contents compared to wild type strain. However, these impacts of *Tgl1* KD were slight when the cells were subjected to the two-stage growth system. The *Tgl1* KD resulted in slight change of fatty acid composition; increasing in C14:0, C16:0 and C16:1, and decreasing in C20:5. This study indicates that, although *Tgl1* played a certain role in lipid degradation in *F. solaris*, suppression of only a single type of TAG lipase was not significantly effective to improve the lipid production. Comprehensive understanding of the lipid catabolism in this microalga is essential to further improve the lipid production.

## Introduction

Depletion of fossil fuels and concerns over climate change have resulted in more intense research into renewable biofuels from microalgae. Microalgae have been recognized as a promising biofuel source due to their ability to produce high lipid content as compared to higher plants. The microalgae contribute to carbon dioxide fixation, and do not compete with food and feed production^1,2^. However, the economic feasibility of using microalgae as a biofuel producer remains challenging as it depends on improving the entire production process^3^. Up to now, a number of researches focused on increasing lipid productivity or blocking the competing pathways in lipid production^4^. However, the engineered strains tended to result in decrease in cell growth^5,6^.

Knockdown (KD) of the genes involved in lipid catabolism, specifically triacylglycerol (TAG) lipases, which catalyze hydrolysis of ester bonds between the glycerol backbone and fatty acids, is one of the possible strategies to increase the lipid accumulation. Remarkably, this approach showed less impact on cell growth in some microalgae including diatoms^7,8^. Among the microalgae species, *Fistulifera solaris* JPCC DA0580, an oleaginous marine diatom, has attracted immense attention as a promising producer of biofuel in particular bio-jet fuel. *F. solaris* has a capability to accumulate high content of lipids (~ 65 w/w%) and shows high growth rate in large scale outdoor cultivation^9–11^. Recently, the whole-genome analysis identified 42 TAG lipase genes in *F. solaris*^10,12^. Among them, the *Tgl1* genes (fso:g13598 and fso:g10609, hereinafter *Tgl1-a* and *Tgl1-b*, respectively), whose homolog was targeted to KD in the model pennate diatom *Phaeodactylum tricornutum*^8^, were highly up-regulated during lipid degradation in *F. solaris*. In contrast, the genes for CGI-58- like lipase (fso:g12351 and fso:g15276), whose homolog was targeted in another model diatom *Thalassiosira pseudonana*^7^, were not significantly up-regulated in *F. solaris*. Finding of two genes for each *Tgl1* and CGI-58- like lipase is due to the allopolyploidy of this diatom believed to be generated by inter-species hybridization^13^. In this study, we decreased the expression of *Tgl1* genes by targeting two copies of homoeologous *Tgl1* in *F. solaris* with an anti-sense approach and assessed its impact on lipid accumulation and growth of this oleaginous diatom. This is the first study to establish the KD technique for allopolyploid microalgae, and also to demonstrate the enhancement of lipid production by TAG lipase KD in the industrially important microalgae applicable for biofuels production.

## Materials and methods

### Strain and culture conditions

The marine diatom *Fistulifera solaris* JPCC DA0580 was routinely maintained in half-strength Guillard’s f medium (f/2) dissolved in artificial seawater (ASW) (Marine art SF-1, Tomita Pharmaceutical Co., Ltd., Tokushima, Japan)^14^. The cells were cultivated at 25 ℃ under continuous light at 130 µmol photons/m^2^/s using fluorescent lamps for plant cultivation Plantlux (Toshiba Co., Tokyo, Japan). The photosynthetic photon flux density was measured using a luminometer HD2302.01 at the wavelength ranging from 400 to 700 nm with a probe LP471PAR (Delta OHM S.r.l, Caselle di Selvazzano, Italy). To check the cell concentrations and oil body volumes of wild type and 4 transformant clones, the diatom cells were cultivated in conical flasks containing 50 mL of f/2 medium (the initial cell concentrations of 1 × 10^6^ cells/mL) at 25 °C under continuous illumination at 50 µmol photons/m^2^/s on the shaker (~ 120 rpm). G-418 (500 μg/mL, an aminoglycoside antibiotic, Merck KGaA, Darmstadt, Germany) was added to the culture of the transformant clones.

The 10f medium which contain tenfold more nutrition components than f medium^15^ was used as nutrient-replete condition, and ASW was used as the nutrient-depleted condition. *F. solaris* wild type and transformants were cultured in the nutrient-replete condition with the initial cell concentration of 1.0 × 10^6^ cells/mL for 72 h in flat-shaped flasks containing 500 mL of 10f medium (the initial cell concentrations of 1 × 10^6^ cells/mL) at 25 °C under continuous illumination at 130 µmol photons/m^2^/s with aeration using sterile air containing 2% CO_2_ at the flow rate of 0.8 L/L/min (vvm) in flat-shaped flasks under the light, temperature, and aeration conditions mentioned above. The cultured cells were collected by centrifugation (8500×*g* for 10 min at 25 °C), and the collected cells were washed twice using ASW, and suspended in ASW (the nutrient-depleted condition), followed by cultivation for 120 h. Cell counts and oil body volume were evaluated at every 24 h.

### Plasmid construction and transformation

The KD vectors targeting two *Tgl1* homoeologous genes, namely *Tgl1-a* (Gene ID: fso:g13598, DDBJ/EMBL/GenBank accession: GAX22768.1) and *Tgl1-b* (fso:g10609. GAX19798.1) were constructed by utilization of pSP-NPT/H4^9^. The antisense fragments, with restriction sites (*Xba*I), were positioned upstream of the terminator region (227 bp) from a fucoxanthin chlorophyll a/c-binding protein A (*fcp*A). The two antisense fragments, *Tgl1* antisense 1 fragment (236 bp) and *Tgl1* antisense 2 fragment (248 bp) (Supplementary Fig. S1), containing 23 bp of effective siRNA fragments (5′-ACA TTG TGA TAG GTT TCT ACC AA-3′ and 5′-CAA TGT CAA TGG CAT GAA TCC AA-3′, respectively) were designed by siDirect v2.0^16^, and amplified by PCR from *F. solaris* genomic DNA. These fragments were then inserted into the *Xba*I site within the expression vector pSP-NPT/H4 to construct pSP-ANT1 and pSP-ANT2, respectively (Supplementary Fig. S2). Supplementary Table S1 shows the list of primers used for construction of these vectors.

The constructed KD vector (pSP-ANT1 or pSP-ANT2) were introduced into *F. solaris* by microparticle bombardment using the Biolistic PDS-1000/He Particle Delivery System (Bio-Rad Laboratories, Inc., Hercules, CA, USA), as described previously^9^. After particle bombardment, the cells (5 × 10^7^ cells per plate) were spread on the f/2 agar medium containing 500 µg/mL of G-418, and incubated at 25 ℃ under the continuous light for 2–3 weeks.

### Quantitative real time PCR

*F. solaris* wild type and the transformants were cultured in flat flasks containing f/2 medium for 72 h and were collected by centrifugation. Total RNA was extracted from the collected cells (1 × 10^8^ cells) using NucleoSpin RNA kit (TaKaRa Bio Inc., Shiga, Japan). cDNA was synthesized with 1 μg of total RNA by Prime Script II 1st strand cDNA Synthesis Kit (TaKaRa Bio Inc., Shiga, Japan). The prepared cDNA (2 ng) was used as the template of quantitative real time PCR (qRT-PCR) with Fast SYBR Green Master Mix (Applied Biosystem, Foster City, CA) and ViiA™ 7 real time PCR system (Life Technologies, Thermo Fisher Scientific, Inc., Waltham, MA, USA). Primers used for qRT-PCR were summarized in Supplementary Table S1. Glyceraldehyde-3-phosphate dehydrogenase (GAPDH) gene was used as a housekeeping gene for normalization of the expression levels with the following equation,

$${\text{Relative expression level of}}\,Tgl1 = \, \left( {{\text{E}}_{{{\text{tgl1}},{\text{transformant}}}} /{\text{ E}}_{{{\text{gapdh}},{\text{transformant}}}} } \right)/\left( {{\text{E}}_{{{\text{tgl1}},{\text{WT}}}} /{\text{E}}_{{{\text{gapdh}},{\text{WT}}}} } \right),$$

where E_tgl1,transformant_ and E_gapdh,transformant_ are expression levels of *Tgl1* and *GAPDH* genes in each transformant, respectively, and E_tgl1,WT_ and E_gapdh,WT_ are expression those in wild type, respectively, analyzed by qRT-PCR.

### Confocal fluorescence microscopy

The microalgal cells stained with BODIPY 505/515 (at least 20 cells of wild type and transformants) were observed using a confocal microscope FLUOVIEW FV1000 (Olympus Corp., Tokyo, Japan). The oil body volumes were calculated by Volocity using confocal microscopic images, as described in previous studies^10,11^.

### Lipid extraction and thin layer chromatography

The cells were incubated in the 10f medium for 72 h to induce lipid degradation, and subsequently lyophilized. The lyophilized cells (15 mg) were suspended in 6 mL of chloroform:methanol (2:1, v/v), and viciously stirred. The mixture was collected by centrifugation at 1000×*g* for 10 min. After the supernatant was collected, the cell pellet was suspended to 3 mL of chloroform:methanol (2:1, v/v), and repeated the above treatment 2 more times. All the supernatant was collected and mixed in the new tube. Next, 1.25 mL of 0.1 M KCl solution was added, and the mixture was collected by centrifugation at 1000×*g* for 10 min. The lower layer of organic solvent was collected, and 10 mg of anhydrous sodium sulfate was added. Finally, the solvent was filtered using the polytetrafluoroethylene (PTFE) syringe filter (0.2 µm, Advantec, Tokyo, Japan) and dried under argon gas.

Extracted total lipids (200 µg) from wild type and transformants cells were spotted onto Glass HPTLC Silica gel 60 plates (Merck Millipore, Massachusetts、U.S.A.), and were separated using petroleum ether: diethyl ether: methanol: acetic acid (90:7:2:0.5, v/v). The detection was performed by exposing the plates to iodine vapor. After spot identification, TAG content was determined by ImageJ^17^. Triolein, diolein, and monoolein (Tokyo Chemical Industry Co., Ltd, Tokyo, Japan) were mixed in the chloroform at the weight ratio of 1:1:1:497 as standards of a triacylglycerol (TAG), diacylglycerol (DAG) and monoacylglycerol (MAG) on the TLC analysis.

For extraction of neutral lipids to estimate the oil productivity, the neutral lipids were extracted using *n*-hexane as described previously^18^.

### Gas chromatography–mass spectrometry

Total lipid extracts of *F. solaris* wild and transformants were transesterified by heating with 1.5 M HCl-methanol at 100 °C for 1 h. After methanolysis, the fatty acid methyl esters (FAMEs) were extracted three times with hexane. The crude extracts were filtered using the polytetrafluoroethylene (PTFE) syringe filter and dried under argon gas. GC–MS-QP2010 Plus (Shimadzu Corporation, Kyoto, Japan) was used to determine the generated FAME compositions. Oven temperatures were programmed at 140 °C for 1 min, then to 240 °C at 4 °C/min and holding at 240 °C for 10 min. FAMEWAX column (30 m, 0.25 mm ID, 0.25 μm, Restek Corporation, USA) was used for separation of FAMEs. A standard fatty acid mixture, FIM-FAME-7 mixture (Matreya, State College, PA, USA) was used. FAMEs were identified by comparing the peak retention times and mass spectra of samples with a standard fatty acid mixture.

## Results and discussion

### Development of the transformants with reduced *Tgl1* expression

**Figure 1:**
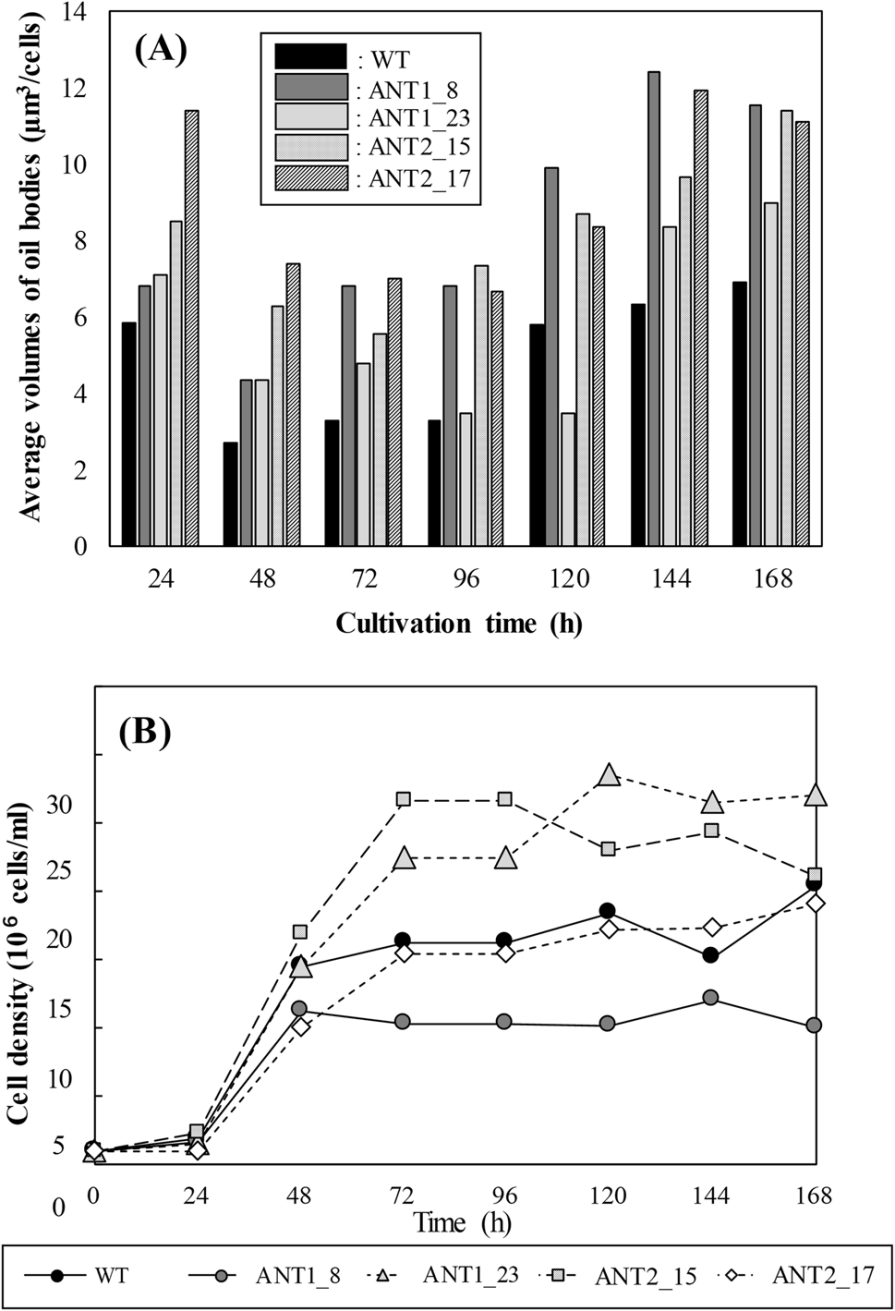
Time-course variation of oil body volumes (**A**) and cell concentrations (**B**) in *F. solaris* wild type (WT) and transformants (ANT1_8 and ANT1_23 for pSP-ANT1, and ANT2_15, and ANT2_17 for pSP-ANT2). The cells were cultured in conical flasks containing 50 mL of f/2 medium (the initial cell concentrations of 1 × 10^6^ cells/mL) at 25 °C under continuous illumination at 50 µmol photons/m^2^/s on the shaker (~ 120 rpm). G-418 (500 μg/mL) was added to the culture of the transformant clones.

KD of *Tgl1* was carried out in *F. solaris* using an antisense approach. The constructs expressing two types of antisense strands (i.e., pSP-ANT1 and pSP-ANT2 vectors) were separately introduced by particle bombardment into *F. solaris*, and 26 and 24 antibiotic-resistant colonies were obtained, respectively. Based on the microscopic observation of the transforming clones, 4 clones (ANT1_8 and ANT1_23 for pSP-ANT1, and ANT2_15 and ANT2_17 for pSP-ANT2) were selected due to the larger oil body volumes than those in wild type at the stationary phase (Fig. 1A). The growth evaluation was performed for these 4 clones to select the clones without growth inhibition caused by the random integration of the vectors into the genomic DNA^9^. As a result, 2 clones (ANT1_23 and ANT2_15) showed higher growth than wild type, while 2 clones (ANT2_17 and ANT1_8) showed comparable (ANT2_17) or even lower (ANT1_8) growth as compared to wild type (Fig. 1B). Transformants harboring the same constructs showed different growth behaviors, and the reason for the detected enhancement of microalgal growth remained unclear. The increased growth pattern in the KD transformants compared to wild type was previously reported for *Thalassiosira pseudonana* with KD of multifunctional lipase (Thaps3_264297). For the subsequent investigation, ANT1_23 and ANT2_15 were selected mainly because these KD transformants showed significantly higher growth than wild type. In addition, these harbored different vectors, and formed larger oil bodies than wild type. We eliminated the clone ANT1_8 and ANT2_17 due to obvious growth inhibition and almost comparable oil body volume to ANT2_15, respectively.

**Figure 2:**
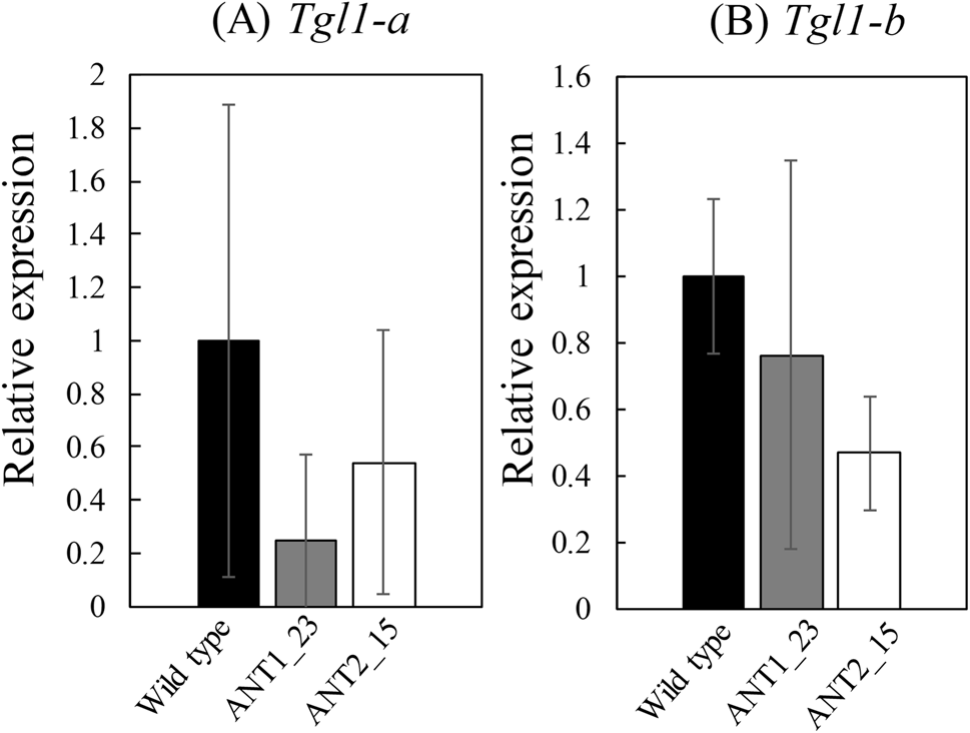
Expression levels of *Tgl1* ((A) *Tgl1-a* and (B)) *Tgl1-b* in *F. solaris* wild type (black bar) and the transformants ANT1_23 (gray bar) and ANT2_15 (white bar) after 72 h cultured in f/2 medium. Transcription abundance was normalized with that of GAPDH gene. Values are means ± SD of three independent cultures.

Subsequently, suppression of *Tgl1* gene expression was confirmed by qRT-PCR. As mentioned above, allopolyploid diatom *F. solaris* has 2 homoeologous *Tgl1* genes (*Tgl1-a* and *Tgl1-b*) that are contained in 2 subgenomes co-existing in a single cell^12^. Thus, to distinguish these genes, expression levels were quantified for each homoeologous *Tgl1* gene using the primer pairs specific to each homoeologous gene. Figure 2 shows the result of the relative expression levels of the target genes *Tgl1-a* and *Tgl1-b*. This result indicates the KD of the target gene of interest in *F. solaris* by expression of antisens fragments was successful. Two antisense fragments tested in this study affected in different manners on the gene suppression. The transformant ANT1_23 expressing antisens1 exhibited substantial suppression of *Tgl1-a* by only 24%, while it showed moderate suppression of *Tgl1-b* by 76%. By contrast, ANT2_15 decreased the expression of both targets by approximately half. Both antisense fragments were designed with 100% sequence match to the mRNA of *Tgl1-a* (Supplementary Fig. S1). Therefore, better suppression of *Tgl1-a* found in the transformant ANT1_23 was in line with the design of antisense1. The reason for comparable suppression of *Tgl1-a* and *Tgl1-b* in the transformant ANT2_15 harboring antisense2 gene remains unclear. Previous studies reported that similar antisense approaches achieved the decrease in the expression of *Tgl1* by 19–45%, and decrease in the expression of another type of lipase *OmTGL* by 6–17% in the model pennate diatom *P. tricornutum*^8,19^. The suppression levels of the lipase genes shown in this study were comparable to those previously reported.

### Attenuation of lipid degradation in the *Tgl1* KD-transformants

**Figure 3:**
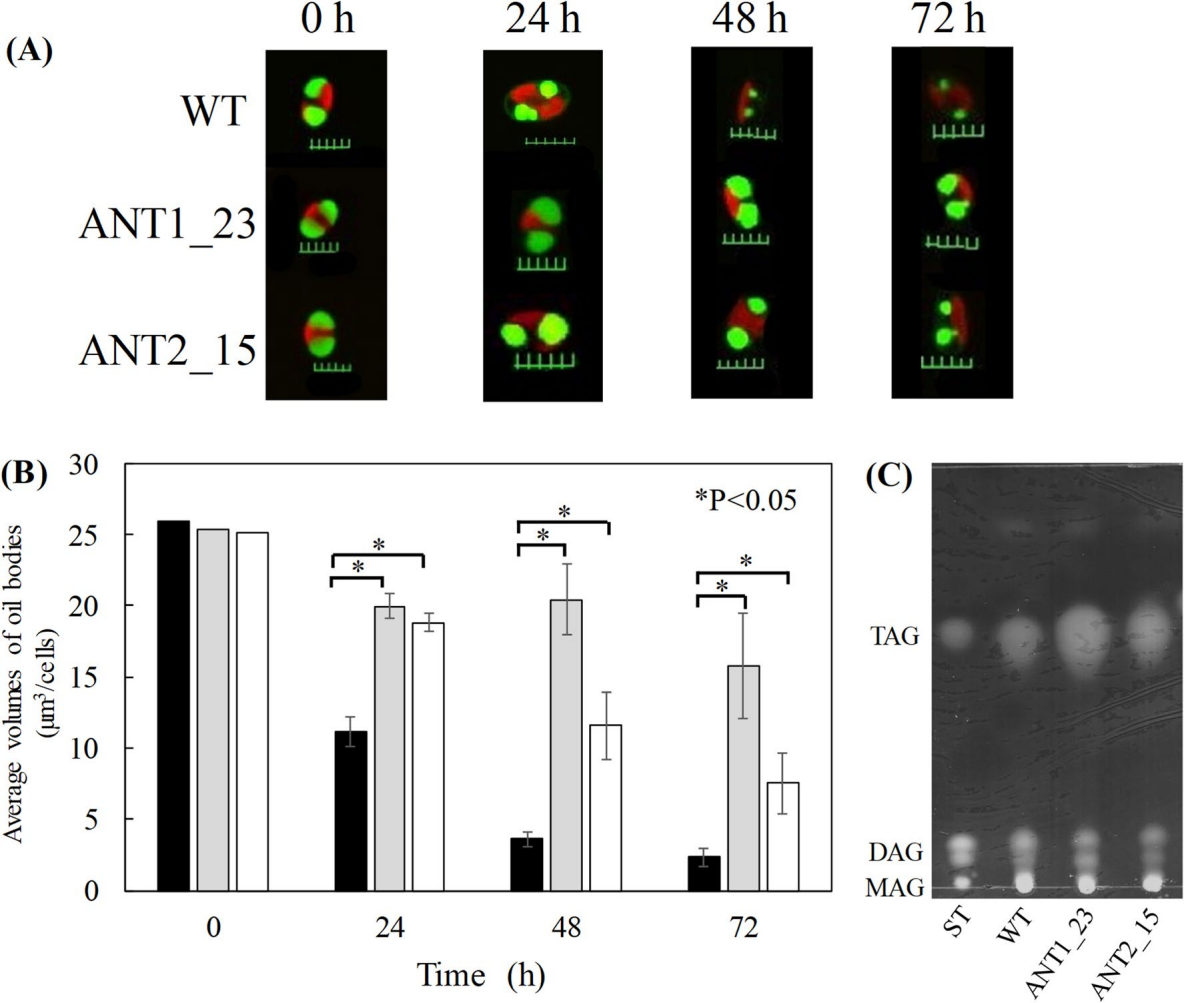
Effect of *Tgl1* KD on lipid accumulation in the diatom *F. solaris*. (**A**) Fluorescent microscopic images of *F. solaris* wild type and the transformants (ANT1_23 and ANT2_15) under nutrient-replete condition. Cells were stained with BODIPY 505/515 (Scale bar = 5 μm). (**B**) Time-course variation of oil body volumes in *F. solaris* wild type (black bar) and the transformants ANT1_23 (gray bar) and ANT2_15 (white bar) under nutrient-replete condition. Welch’s *t*-test was used to compare two groups. Difference was assessed with two-side test with an alpha level of 0.05. Values are means ± SD of 20 independent cells. (**C**) TLC analysis of the lipid extracts of *F. solaris* wild type (WT) and the transformants ANT1_23 and ANT2_15 at 72 h cultured under nutrient-replete condition. Lipid standards (ST): triolein (TAG); diolein (DAG); monoolein (MAG). The cropped image of the TLC plate is shown in this figure, and the uncropped and not-inverted image of the plate is presented in Supplementary Fig. S3.

Lipid remobilization involves in three majors temporally and spatially steps^20^. First, the degradation of TAG to fatty acid by lipase protein. Next, β-oxidation process is occurred to produce acetyl-coA, which finally involved in tricarboxylic acid (TCA) to produce energy. In our study, we focused on the first step of the lipid remobilization in order to investigate the KD of *Tgl1* lipase in *F. solaris*. Therefore, we utilized the term “degradation” rather than “remobilization”. To determine the attenuation of lipid degradation in *Tgl1* KD-transformants (ANT1_23 and ANT2_15), degradation of neutral lipids in the cells were induced by transferring the cells from the nutrient deplete condition to nutrient replete condition. During the lipid degradation induced by nutrient-supplementation, fluorescence images of the cells with BODIPY-staining were obtained to evaluate the oil body volumes. It should be noted that our previous studies already reported that oil body volumes and neutral lipid contents were highly associated^10,11^. As a result, oil body volumes of these 3 strains were comparable at 0 h (Fig. 3A,B). As compared to the time at 0 h, oil body volumes decreased in all strains, while the transformant clones ANT1_23 and ANT2_15 maintained larger oil bodies in the cells under a lipid degradation condition at 72 h with the oil body volumes 15.8 and 7.5 μm^3^/cells, respectively (Fig. 3B). Oil volume in ANT1_23 and ANT2_15 were approximately 6.8 and 3.2-fold higher than wild type. Based on the TLC result (Fig. 3C, Supplementary Fig. S3), TAG, DAG and MAG were the main component of the extracted lipids. The TAG content by TLC was 2.8 times higher in ANT1_23 than in the wild-type strain, and 1.8 times higher in ANT2_15 than in the wild-type strain. The results together suggest that *Tgl1* genes targeted in this study were involved in the lipid degradation. Difference in the attenuation effect of lipid degradation between ANT1_23 and ANT2_15 could be attributed to the gene suppression manners. In the clone ANT1_23, *Tgl1-a* was strongly suppressed rather than *Tgl1-b*, while both genes were moderately suppressed in the clone ANT2_15. This data imply that *Tgl1-a* might play a major role in neutral lipid degradation under the tested condition. Indeed, our previous study reported that *Tgl1-a* were highly up-regulated compared to during the lipid degradation process^10^. In allopolyploid genome of *F. solaris*, two pseudo-parental subgenomes (Fso_h and Fso_l) were classified based on the analysis of GC contents in the protein-coding regions^21^. The *Tgl1-a* and *Tgl1-b* genes belong to the subgenomes Fso_h and Fso_l, respectively, and *Tgl1-a* in Fso_h was likely more contribute to lipid degradation than *Tgl1-b*. This notion was consistent with our previous prediction that the genes in Fso_h subgenome might be more involved in lipid degradation-related metabolism than those in Fso_l subgenome, based on the global transcriptome analyses^13^.

### Fatty acid composition in the *Tgl1* KD strain

**Figure 4:**
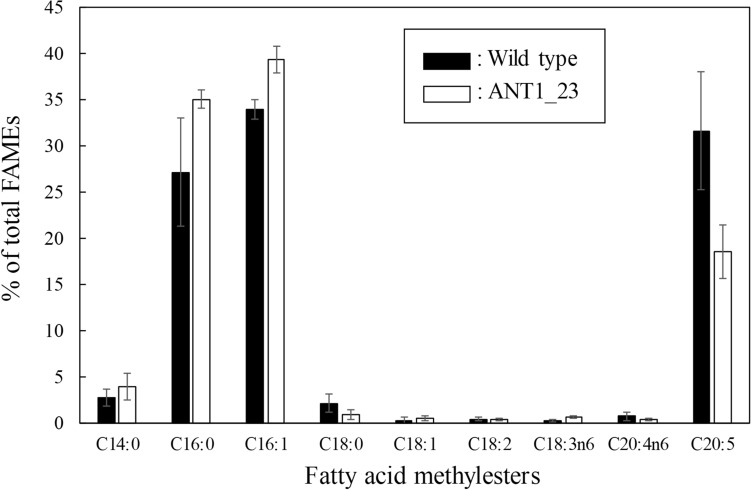
Fatty acid compositions of *F. solaris* wild type and transformant (ANT1_23) at 72 h cultured under nutrient-replete condition. Values are means ± SD of three independent cultures.

The fatty acid compositions of the total lipids in wild type and ANT1_23 were analyzed by GC–MS. We revealed that those were similar in *Tgl1* KD-transformant ANT1_23 and wild type in which palmitic acid (C16:0), palmitoleic acid (C16:1), and eicosapentaenoic acid (EPA, C20:5) occupied the major proportions of the fatty acids (Fig. 4). The most abundant fatty acids were C16:0 and C16:1 together contributed up to 60% of the total lipid. Most prominently, the content of C14:0, C16:0 and C16:1 increased in *Tgl1* KD-transformant ANT1_23, but decreased in C20:5 content compared to wild type. Similar result was found in *Tgl1* KD gene of the model diatom *P. tricornutum*^9^. However, the molecular mechanism underlying this phenomenon was not elucidated. A possible explanation for this phenomenon would be the substrate specificity of the suppressed *Tgl1* lipases, which might prefer to hydrolyze the ester bonds between the glycerol backbone and palmitic acid or palmitoleic acid moieties according to the fatty acid composition of *F. solaris* transformant ANT1_23. The different result in the study by Li et al., found that the content of C16:0 in total lipid decrease while the content of C20:5 increase in *OmTGL* mutant in *P. tricornutum* compared to wild type. In addition, KD of phospholipase A2 in *Chlamydomonas reinhardtii* shows decrease in fatty acid composition of C16 and C18:3 compared to wild type^22,23^. Different result in our study most probably causes by different substrate specificity of different lipase.

### Effect of lipid accumulation in *Tgl1* KD strain

**Figure 5:**
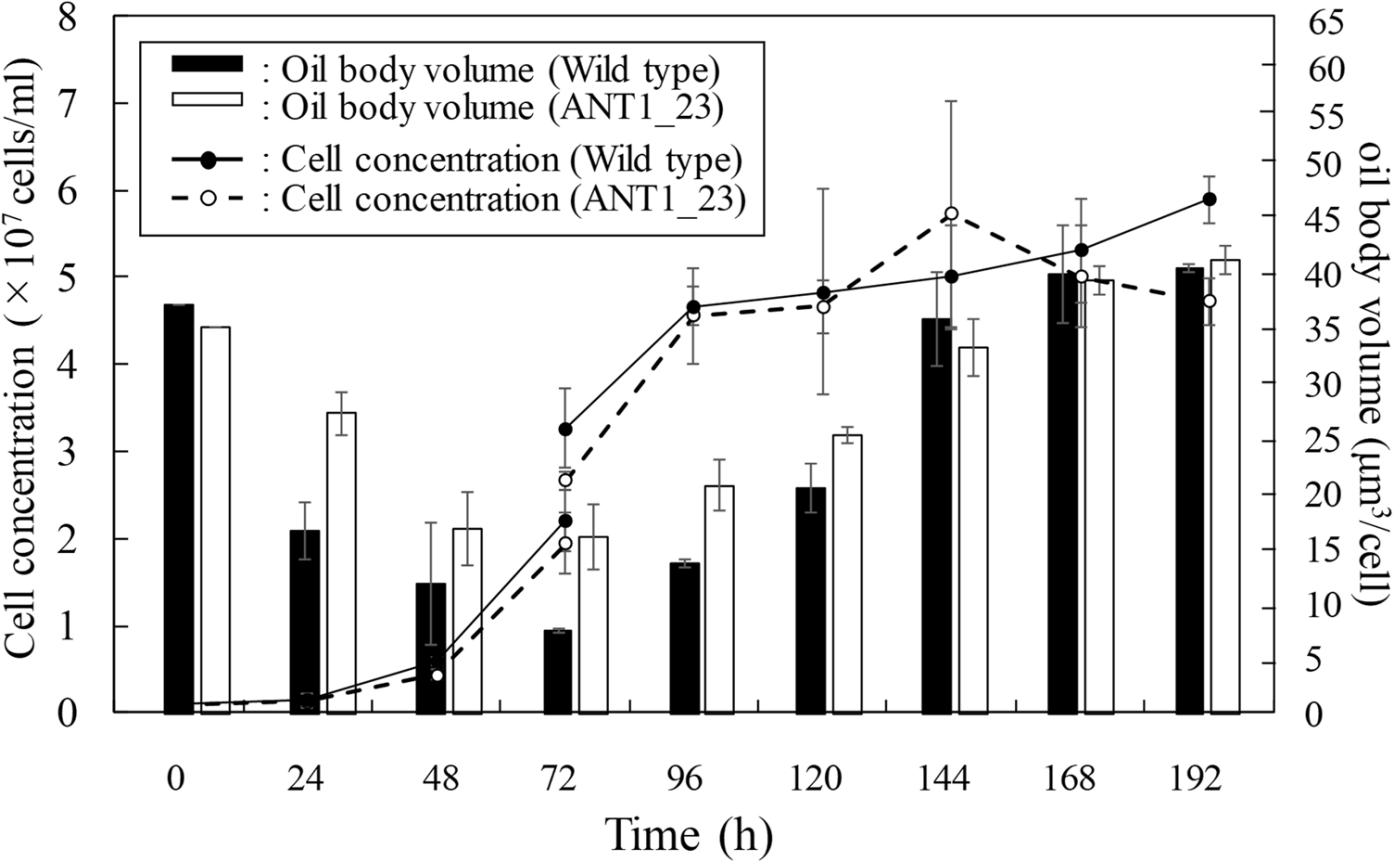
Time-course variation of cell concentration and oil body volumes in *F. solaris* wild-type (WT) and transformant ANT1_23 in two-stage growth system. Cultured cells were transferred to the nutrient-deplete medium at 72 h. Values are means ± SD of three independent cultures.

We designed the improved bioprocess for lipid production with the *Tgl1* KD-transformant ANT1_23, because it maintained higher amount of lipids than wild type and ANT2_15 even under the lipid degradation condition (Fig. 3B). Two-stage growth system (a nutrient repletion stage for massive cell growth for 72 h, followed by a nutrient depletion stage to induce lipid accumulation) was employed because this cultivation condition maximized the lipid productivity^24–26^. The growth was comparable between wild type and *Tgl1* KD transformant ANT1_23 under this cultivation condition (Fig. 5). The oil body volumed in both *Tgl1* KD transformant ANT1_23 and wild type decreased when subjected to nutrient repletion condition for 72 h (Fig. 5). When compared, the oil body volumes in ANT1_23 was significantly higher than wild type because the impaired degradation of storage lipids enhanced the TAG content in replete cells. This result clearly demonstrated the impact of *Tgl1* KD. Subsequently, the cells were transferred to the nutrient depletion stage. The lipid accumulation increased in *Tgl1* KD transformant ANT1_23 and wild type strains. *Tgl1* KD transformant ANT1_23 showed 1.2 times higher lipid accumulation than wild type until 120 h (48 h after nutrient depletion). However, the maximal capacities of lipid accumulation of those at 192 h were comparable. Little change in the maximal lipid capacities between wild type and *Tgl1* KD transformant ANT1_23 could be caused by the limited intracellular spaces. Therefore, KD of *Tgl1* in the oleaginous diatom showed limited impact on the cell growth and lipid accumulation in the two-stage growth system (Fig. 5). These results suggest that lipid catabolism in the diatom might be more complex, and could not be solely catalyzed by *Tgl1* lipases, although *Tgl1* likely played a certain role in TAG degradation. Our previous study reported that autophagy-mediated lipid degradation (lipophagy) could be involved in lipid catabolism in *F. solaris*^27^, suggesting that KD of the genes related to lipophagy can be another strategy to increase the storage lipids.

## Conclusions

In this study, KD of *Tgl1* genes was performed by an antisense approach to improve oil productivity in marine diatom *Fistulifera solaris* JPCC DA0580. Our result indicated that KD transformants enhanced cell growth and lipid accumulation under the conventional cultivation process. The *Tgl1* KD cells even under the oil degradation-induced condition maintained up to 2.8-fold higher oil content than wild type. However, no significant difference was found in the maximal lipid capacities between KD mutant and wild type under two-stage growth system (nutrient repletion for 72 h followed by nutrient depletion condition). This result showed that the impacts of *Tgl1* KD on the growth and lipid accumulation under two-stage growth system were limited despite it showed positive impact under normal cultivation. A deeper understanding on lipid degradation pathway in diatom is necessary to improve lipid productivity.

## Supplementary Information

Supplementary Information.
