# Supplementary material for: Assessment on the oil accumulation by knockdown of triacylglycerol lipase in the oleaginous diatom Fistulifera solaris
Source: Sci Rep. 2021 Oct 22;11:20905. doi: 10.1038/s41598-021-00453-w (PMC8536745; doi:10.1038/s41598-021-00453-w)
Supplement: Supplementary file 1 — Supplementary Information. [file 41598_2021_453_MOESM1_ESM.pdf]

Supplementary Information for:

Assessment on the oil accumulation by knockdown of triacylglycerol lipase in the oleaginous diatom *Fistulifera solaris*

Yoshiaki Maeda[a], Kahori Watanabe[a], Marshila Kaha[a], Yusuke Yabu[a], Tomoko Yoshino[a], Mitsufumi Matsumoto[b], Tsuyoshi Tanaka[a*]

[a]Division of Biotechnology and Life Science, Institute of Engineering, Tokyo University of Agriculture and Technology, 2-24-16 Naka-cho, Koganei, Tokyo 184-8588, Japan,

[b] Biotechnology Laboratory, Electric Power Development CO., Ltd., 1, Yanagisaki-machi, Wakamatsu-ku, Kitakyushu 808-0111, Japan

*Corresponding author

E-mail address: tsuyo@cc.tuat.ac.jp

Tel: +81-42-388-7401

Fax: +81-42-385-7713

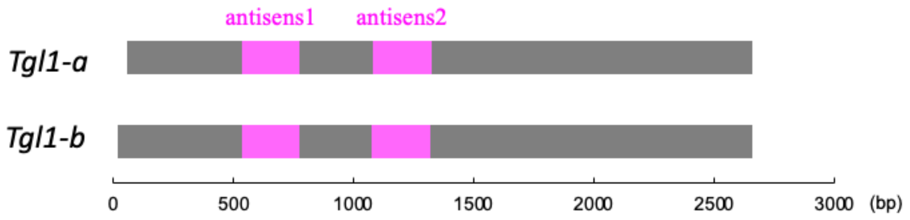

[antisens1]

```
Tgl1-b       AGTATTGGACGCAATAAATTTGGTCTCTTACATGAAGGATTGTTCACCAAAGCACGGGCC   540
Tgl1-a       AGTATTGGACGCAATAAATTTGGACTCCTTCATGAAGGATTGTTTACCAAGGCCCGGGCC   483
antisens1    ----------------------------------------------------------CC   2
                                                                       **

Tgl1-b       GGTACCAAAGTCTTGGTTGAAACCTATCACAATGTCATTTGTGCCGCTTTGGATTTTGTC   600
Tgl1-a       GGTACCAAAGTCTTGGTAGAAACCTATCACAATGTCATTTGTGCCGCCTTGGATTTTGTC   543
antisens1    GGTACCAAAGTCTTGGTAGAAACCTATCACAATGTCATTTGTGCCGCCTTGGATTTTGTC   62
             ***************** ***************************** ************

Tgl1-b       TGTGATGCACAAGTCTTACCGGATCAAGATCCGATTCCCACCGAAGCACGATTGGCTTTT   660
Tgl1-a       TGTGATGCCCAAGTGTTACATGATCAAGATCCGATTCCCACTGAAGCACGATTGGCCTTT   603
antisens1    TGTGATGCCCAAGTGTTACATGATCAAGATCCGATTCCCACTGAAGCACGATTGGCCTTT   122
             ******** ***** ****  ******************* ************** ***

Tgl1-b       TTCAATGAAACACGCCATTCGTATGGTCGGACCGCTCTCTTGTTGAGTGGTGGAGCCGCC   720
Tgl1-a       TTCAATGAAACACGACATTCGTACGGTCGGACGGCGCTCTTGTTGAGTGGCGGAGCCGCC   663
antisens1    TTCAATGAAACACGACATTCGTACGGTCGGACGGCGCTCTTGTTGAGTGGCGGAGCCGCC   182
             ************** ******** ******** ** ************** *********

Tgl1-b       TTGGGATTTTATCATGTCGGTGTCGTCCGGACCTTGATGGAAAATCGTCTCATGCCCCGC   780
Tgl1-a       TTGGGATTTTATCACGTCGGTGTGGTCCGGACCTTGATGGAAAATCGTCTCATGCCCCGG   723
antisens1    TTGGGATTTTATCACGTCGGTGTGGTCCGGACCTTGATGGAAAATCGTCTCATG------   236
             ************** ******** ******************************
```

[antisens2]

```
Tgl1-b       GCAGCCACGTGGTTGGATTTCTATTACAACACGGCCGGTGCCTTTCATGATATGAAATTG   1074
Tgl1-a       ACAGCCTCGTGGTTGGATTTCTATTACAACACGGCCGGTGCCTTTCATGATATGAAATTG   1023
antisens2    -----------------------------------------------------------G   1
                                                                        *

Tgl1-b       ACATTGATTGGATTCATGCCATTGACATTACGACGCATGACGGCGTTCTTGTATGACGTT   1134
Tgl1-a       ACATTGATTGGATTCATGCCATTGACATTGCGACGCATGACAGCGTTCTTGTATGACGTC   1083
antisens2    ACATTGATTGGATTCATGCCATTGACATTGCGACGCATGACAGCGTTCTTGTATGACGTC   61
             ***************************** *********** *****************

Tgl1-b       TTGTCGGGAAGTCGACGGGCTTCCGATGTCTTTAAGAAGGATACCGACTATTTTCGCGAA   1194
Tgl1-a       TTATCGGGCAGTCGACGAGCGTCGGATGTCTTTAAGAATGATACCGATTATTTTCGCGAA   1143
antisens2    TTATCGGGCAGTCGACGAGCGTCGGATGTCTTTAAGAATGATACCGATTATTTTCGCGAA   121
             ** ***** ******** ** ** ************** ******** ************

Tgl1-b       TGTGTTCGCGCCAATGTGGGAAACTTTACATTTCAAGAAGCCTTTGATCGGACGGGACGT   1254
Tgl1-a       TGCGTCCGCGCCAATGTGGGAAATTTTACCTTTCAAGAAGCCTTTGATCGGACGGGACGT   1203
antisens2    TGCGTCCGCGCCAATGTGGGAAATTTTACCTTTCAAGAAGCCTTTGATCGGACGGGACGT   181
             ** ** ***************** ***** ******************************

Tgl1-b       ATTTTGAATATTGTCGTGACACCCATGAATAGCAGTGATCCGCCGCGTTTGCTCAATTAC   1314
Tgl1-a       ATTTTGAACATTGTCGTGACACCCATGAATAGCAGTGACCCGCCGCGTTTGCTCAATTAC   1263
antisens2    ATTTTGAACATTGTCGTGACACCCATGAATAGCAGTGACCCGCCGCGTTTGCTCAATTAC   241
             ******** ***************************** *********************

Tgl1-b       TTGACGGCACCGCATGTCATGGTATGGAGTGCCGCCGTGGCCAGTAGTTCTTTGCCTGGT   1374
Tgl1-a       TTGACGGCACCGCATGTTATGGTCTGGAGTGCTGCCGTGGCCAGCAGTTCGTTGCCCGGT   1323
antisens2    TTGACGG-----------------------------------------------------   248
             *******
```

Fig. S1 Sequence alignments of *Tgl1* gene (*Tgl1-a* and *Tgl1-b*) and antisense fragments.

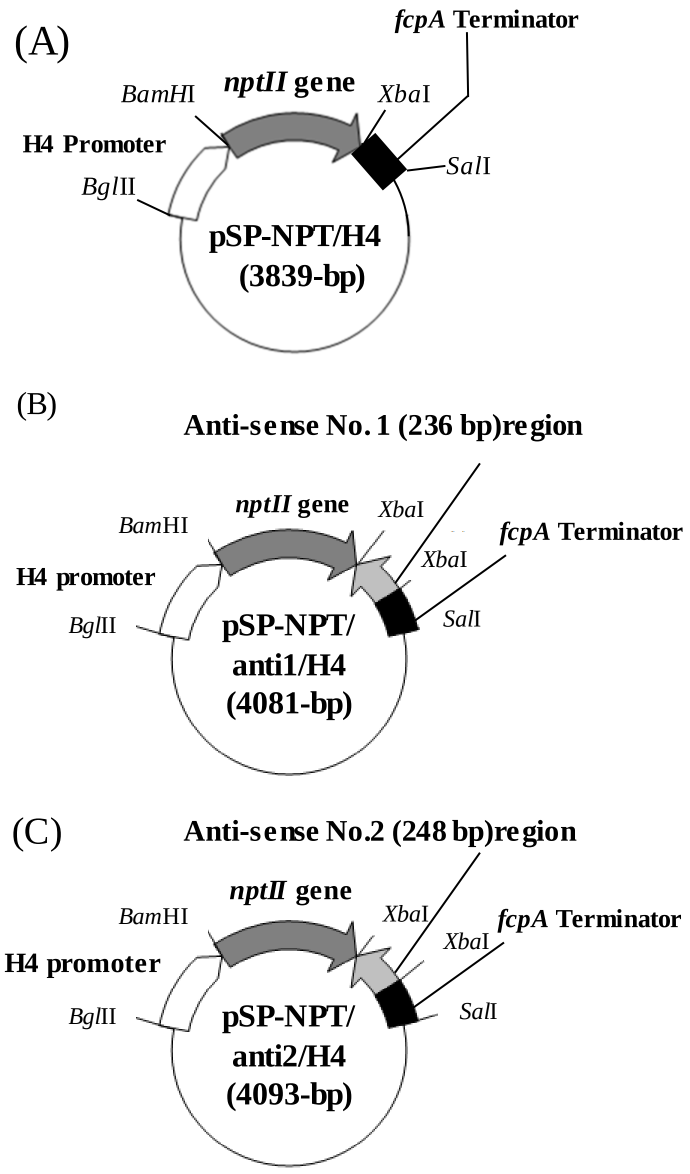

Fig.S2 Constructions of vectors used in this study. Restriction maps of the expression cassettes in plasmids pSP-NPT/H4 (A), pSP- ANT1 (B), and pSP-ANT2 (C).

Abbreviations: *H4* prmoter, histone H4 gene promoter from *F. solaris; fcp A* terminator, fucoxanthin chlorophyll *a/c*-binding protein A gene terminator from *P. tricornutum*.

Table S1 Primers used in this study

Primer name	Sequence (5'-3')	Use
Tgl1_anti_2F	AAATTTTCTAGACATGAGACGATTTTCCATCA	Cloning by restriction enzyme
Tgl1_anti_2R	AAATTTTCTAGACCGGTACCAAAGTCTTGG	
Tgl1_anti_3F	AAATTTTCTAGACCGTCAAGTAATTGAGCAAA	
Tgl1_anti_3R	AAATTTTCTAGAGACATTGATTGGATTCATGC	
anti2_fwd	TGATCTAGACATGAGACGATTTTCCATC	Cloning by Gibson assembly
anti2_rev	GTTTCTAGACCGGTACCAAAGTCTTGG	
anti2-1_fwd	GGTACCGGTCTAGAAACAACTACCTCGACTTT	
anti2-1_rev	CGGAGGCAGATCTGGTTCCCGCATAG	
anti2-2_fwd	ACCAGATCTGCCTCCGGTACTCTTAC	
anti2-2_rev	GTCTCATGTCTAGATCAGAAGAACTCGTC	
anti3_fwd	TGATCTAGACCGTCAAGTAATTGAGCAAAC	
anti3_rev	GTTTCTAGAGACATTGATTGGATTCATGC	
anti3-1_fwd	ATCAATGTCTCTAGAAACAACTACCTCGACTTT	
anti3-1_rev	CGGAGGCAGATCTGGTTCCCGCATAG	
anti3-2_fwd	ACCAGATCTGCCTCCGGTACTCTTAC	
anti3-2_rev	CTTGACGGTCTAGATCAGAAGAACTCGTC	
anti_seq_F	GAATATCATGGTGGAAAATGG	Confirmation of introduction of antisense sequence
anti_seq_R	CATGGTGAAGACGAGCTAGT	
RNAi_nptII_F	TGAACAAGATGGATTGCAC	Sequence analysis of Neomycin casette
RNAi_nptII_R	CAGAAGAACTCGTCAAGAAGG	
qPCR_GAPDH_F	ATTGGAGTCAATGGCTTTG	Quantitative RT-PCR to determine quantity of GAPDH gene expression level
qPCR_GAPDH_R	CCATGAACGGAATCGTACT	
qPCR_g13_anti2F	TTGGACTCCTTCATGAAGG	Quantitative RT-PCR to determine quantity of Tgl1 gene expression level
qPCR_g13_anti2R	GATCATGTAACACTTGGGCA	
qPCR_g13_anti3F	CATTCCAGTACCGTCACG	
qPCR_g13_anti3R	GACTGCCCGATAAGACGT	
qPCR_g10_anti2F	ATTGTTCACCAAAGCACG	
qPCR_g10_anti2R	GATCCGGTAAGACTTGTGC	
qPCR_g10_anti3F	CCATAGTACAGTCACGACCTC	
qPCR_g10_anti3R	GACTTCCCGACAAAACGT	

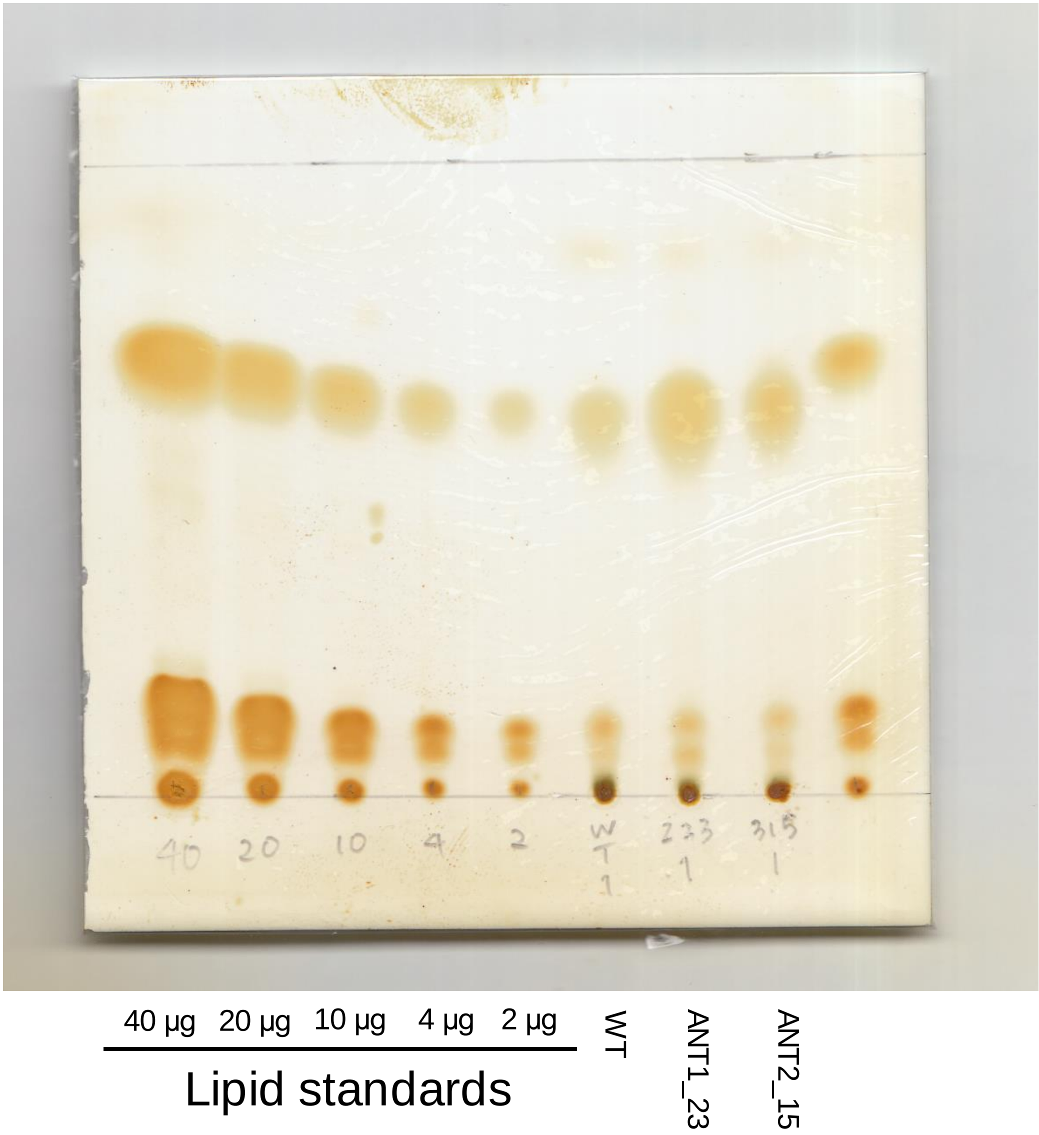

Fig. S3 Uncropped and non-inverted full-length image of the TLC plate shown in Figure 3 in the main text.
